# Post-mortem ultrasound study of coronary circulation on ex-situ hearts

**DOI:** 10.1007/s00414-025-03683-z

**Published:** 2025-12-16

**Authors:** Simone Santelli, Marcello Ridolfi, Filippo Pirani, Elena Giovannini, Guido Pelletti, Giovanni Cecchetto, Susi Pelotti, Paolo Fais

**Affiliations:** 1 Department of Medical and Surgical Sciences, Unit of Legal Medicine, University of Bologna, Via Irnerio 49, Bologna, 40126 Italy; 2 Pediatric and Adult CardioThoracic and Vascular, Oncohematologic and Emergency Radiology Unit, IRCCS Azienda Ospedaliero-Universitaria di Bologna, Bologna, Italy; 3 Department of Legal Medicine and Forensic Sciences, University of Pavia, Pavia, Italy

**Keywords:** Ultrasound, Sudden cardiac death, Coronary arteries, Coronary pathology, Post-mortem imaging, Imaging in forensics

## Abstract

Sudden cardiac death is a leading cause of death in the 40–65 age group in industrialized countries. In approximately 70–80% of cases, it is attributable to atherosclerotic degeneration of the coronary circulation. This experimental study aims to evaluate the suitability of ultrasound for the morpho-functional characterization of the coronary circulation in ex situ hearts. The coronaries of six human hearts were cannulated with a vascular catheter, and an aqueous contrast medium was injected using an electric pump. The cannulated vessel underwent ultrasound scanning (US) for both morphological and functional study using US and color Doppler. Subsequently, a cardiopathological examination of each heart was performed. US and color Doppler allowed the characterization of the coronary vessels, the morphology of atherosclerotic plaques, the degree of vascular stenosis, and related dysfunctional, reduced or turbulent flow. Subsequent cardio-pathological examination confirmed the US findings. Preliminary results indicate coronary US as a promising diagnostic technique in cardio-pathology, as a preliminary screening test for subsequent targeted investigations, since it enables the pathologist to assess the dysfunctional implications of atherosclerotic disease on coronary flow. Future perspectives include extending the studied sample and comparing post-mortem US with other techniques commonly used for the study of coronary pathology.

## Introduction

Ultrasound (US) is a non-invasive, widely used diagnostic tool that allows the visualization of any section of the body through the use of mechanical waves and echoes. The evaluation of echotexture of tissues allows distinguishing healthy organs from pathologic processes. It is also defined as a “real-time” diagnostic tool, since structures can be seen moving in the image (e.g., cardiac valves). It is highly accurate in the evaluation of superficial structures and it is widely used for the visualization of soft tissues and vessels. An additional advantage of US is the exploitation of the Doppler effect (color Doppler US), which gives information about the speed and direction of the moving structure in the image, particularly used in evaluating blood vessels and blood flow. The velocity of red blood cells allows an estimation of the vessel diameter : as the vessel lumen narrows, generally the velocity of red cells increases. These data are used to assess areas of arterial narrowing/stenosis (e.g., carotid stenosis) and to determine if masses and organs have an increased blood flow [1, 2].

Although it is a very user-dependent process, US is considered a valuable tool in clinical settings, for the diagnosis of a wide range of different pathologies and is considered highly reliable for the diagnosis of vascular pathologies of superficial vessels, both peripheral and central, and in fact some of the main arteries of interest, such as the carotid arteries, are routinely assessed with the US [3]. The US has been tested and documented as a valuable tool for the non-invasive assessment of coronary vessels in living patients since 1976, with good results [4], and its use has been tested also in specific settings, such as in the evaluation of coronary malformations in pediatric patients [5–11].

Despite its many advantages in clinical settings, evidence for the usefulness of post-mortem US in forensic practice is still scarce [12, 13]. The uses reported for US in post-mortem imaging are mainly related to fetal and perinatal deaths, pediatric diseases [14], guided tissue sampling (especially in the suspect of an infectious disease and widely used during the COVID-19 pandemic) [15, 16], and measurement of rigor mortis through US elastography for post-mortem interval estimation [17, 18]. To the best of our knowledge, the application of ultrasound in cases of suspected sudden cardiac death (SCD) has not been explored within the field of forensic pathology.

Currently, the gold standard for the assessment in cases of suspected SCD is the autopsy followed by a complete pathological evaluation of the heart, including macroscopic and microscopic analysis [19]. Several post-mortem imaging techniques, such as the computed tomography (CT) angiography [20], Magnetic Resonance Imaging (MRI) and thermal angiography [21, 22] have been proposed.

The aim of the present pilot study is to test the suitability of the US and of the color Doppler for post-mortem assessment of coronary arteries (CA) on ex-situ human hearts through the development and application of an artificial, post-mortem coronary circulation.

## Materials and methods

The study was approved by the Bioethics Committee of the University of Bologna (protocol no. 0154004). The study was conducted on six adult human hearts that had been extracted during conventional medicolegal autopsy following the local standard autopsy procedure (extraction of the heart and origins of the aorta, pulmonary artery, and veins after opening the pericardium). The time from death to coronary evaluation varied from 24 to 48 h.

### Ultrasonography

Two different US and color Doppler US devices were used, namely an ESAOTE MyLab Xpro80 with a high-frequency linear probe L4-15 MHz and a TOSHIBA Aplio 500 with a high-frequency linear probe PLT-1005BT 10 MHz. The vascular study presets used during Doppler assessments were primarily set with the following parameters:

- Sample volume (SV) 2/23 mm;
- Insonation angle between 50° and 60°;
- Pulse Repetition Frequency (PRF) 1.5 kHz;
- Frequency scale (frq) 0.13 m/s;
- Velocity scale (vel) 30 / -10 cm/s.

### Preparations of the hearts and cannulation of coronary arteries

**Fig. 1:**
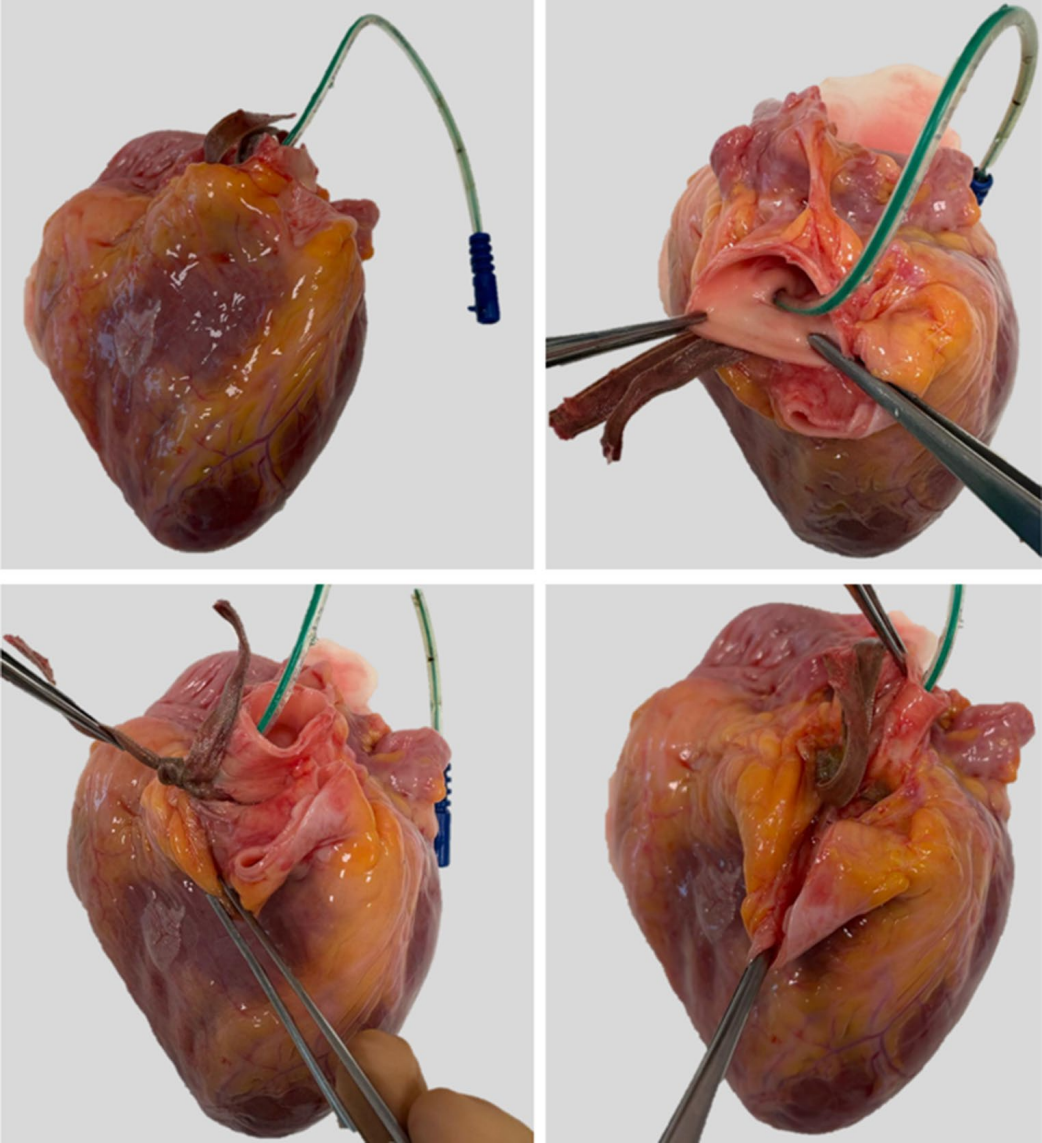
Cannulation of the right coronary artery. The proximal segment ofthe artery was secured with a satin band, tied using an orthodontic rubber band

According to a previous angiography study [22], the root of the aorta and the pulmonary artery were separated down to their proximal portion and a segment of the aorta was removed with a transverse cut in order to expose the three aortic cusps into the remaining stump of the ascending aorta. The orifice and origin of the right and left CAs were identified. Using a central venous catheter for dialysis for equine hearts (Niagara™ Slim-Cath^®^) and a Ch 6 infant feeding tube for human hearts (Unomedical—ConvaTec limited, Deeside, UK), the coronary ostia were gently cannulated and the course of the vessels was followed (Fig. 1). To ensure that the catheter was maintained in place, the proximal tract of the vessel was dissected and isolated, then a ligature with a 0.5-cm-wide satin band and an orthodontic rubber band was performed (Fig. 1).

### US and color Doppler US

Two expert radiologists performed independently US and color Doppler US scans on all the hearts in separate sessions. At first, the US examination was performed for the morphological assessment of each heart. Subsequently, the catheter was connected via a 10 cm rubber tube to an electric pump (ANRNS, maximum flow rate 180 L/H), which was used to inject water into the cannulated vessel. After activating the pump another US scan was conducted on each heart for the morphological assessment during the continuous injection of water. In particular, atherosclerotic lesions and the degree of vascular stenosis were assessed within each major coronary artery.

 After the morphological assessment, the same radiologists performed the functional evaluation of the coronary circulation through color Doppler imaging. During the Doppler scans, the electric pump was activated to create an artificial circulation allowing Doppler imaging to detect the direction and velocity of flow in both healthy coronary artery segments and those affected by atherosclerotic plaques. To further assess the modifications of the water flow into the vessel, in the final part of the experiment the more distal segment of the coronary artery was cut transversely with a scalpel to reduce flow resistance.

### Examination of coronary arteries

After the complete US assessment of the coronary circulation, the hearts were formalin fixed and underwent a complete pathological examination according to current guidelines [19]. In particular, coronary examination was performed by an expert pathologist through multiple transverse cuts at 3-mm intervals along the course of the main epicardial arteries, including branches such as the diagonal and obtuse marginal, and the lumen was evaluated. The pathological examination focused on the same coronary sections previously assessed by US. Formerly, the coronary lumen area measured by US was compared to the area determined from both macroscopic and microscopic analysis. Then, within the atherosclerotic segments, morphological features identified via US were correlated with the corresponding macroscopic and histopathological findings.

## Results

### Morphological study

**Fig. 2:**
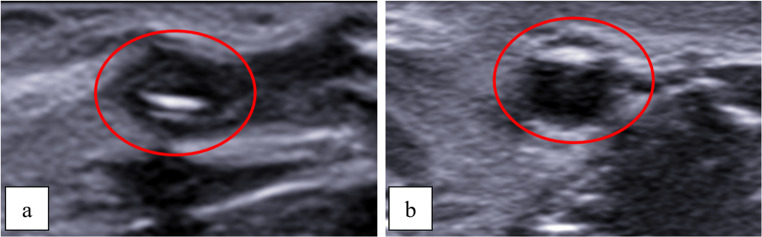
Transverse scan. Prior to activation of the electric pump, the vessels (red circle) were collapsed, and the lumen was not visible (**a**). Following pump activation, water flow distended the vessels (red circle), allowing full visualization of the lumen (**b**)

 In the studied sample, the US scan allowed the radiologist to visualize the epicardial and myocardial tissues, although the coronary vessels were collapsed and both walls and lumen were difficult to detect (Fig. 2a). On the other hand, after the activation of the pump simulating the coronary circulation, the US provided a good visualization of the vessels (Fig. 2b).

**Fig. 3:**
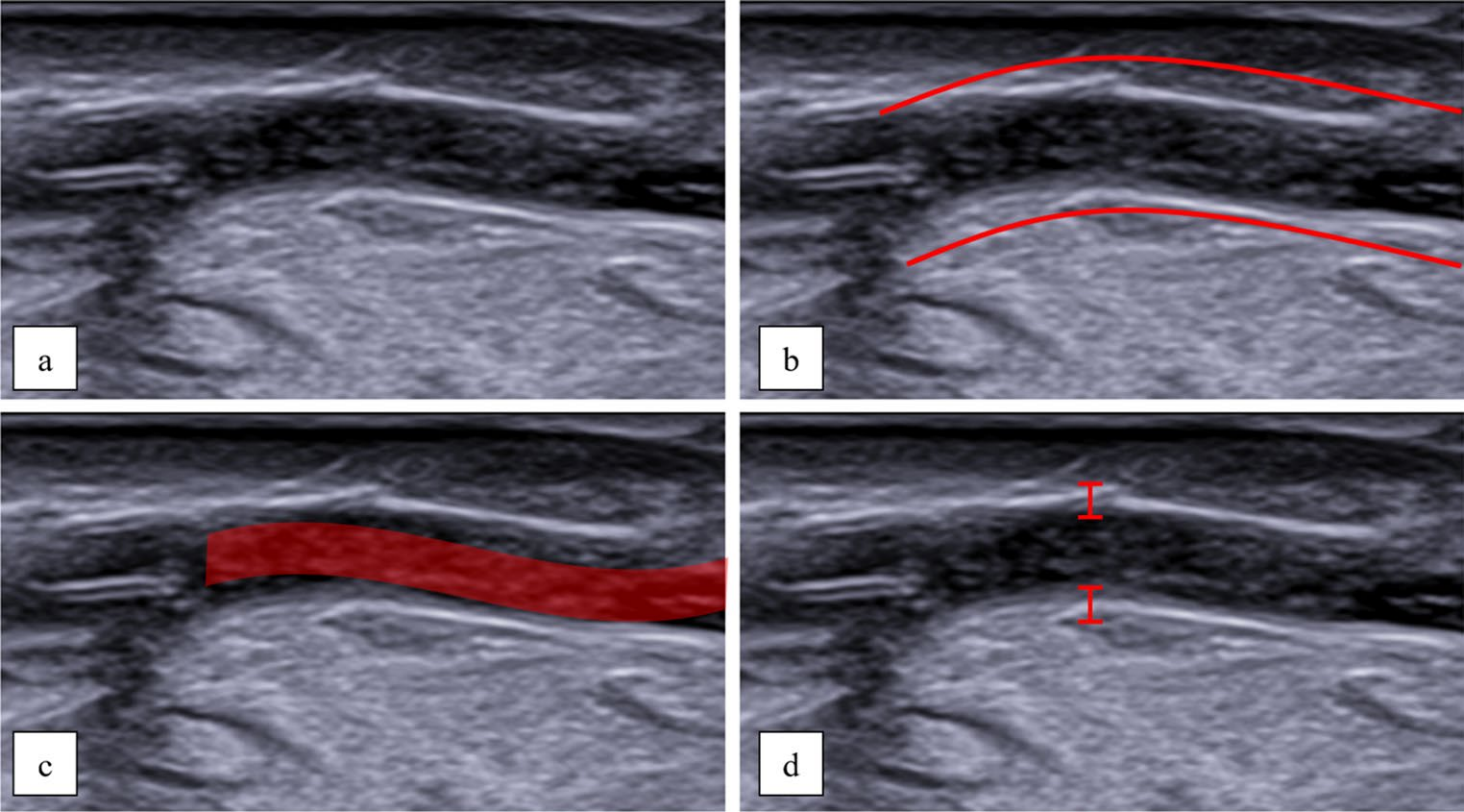
Longitudinal scan. Following activation of the electric pump, the vessel was distended and clearly visible (**a **and **b**). Water flow within was apreciable (**c**), and the vessel walls were clearly delineated (**d**)

 More specifically, activating the electric pump allowed for the visualization of the coronary lumen and walls, as well as the internal fluid flow after the activation of the electric pump, it was possible to visualize the lumen and the vascular walls of the coronaries as well as the waterflow within the vessel itself (Fig. 3).

**Fig. 4:**
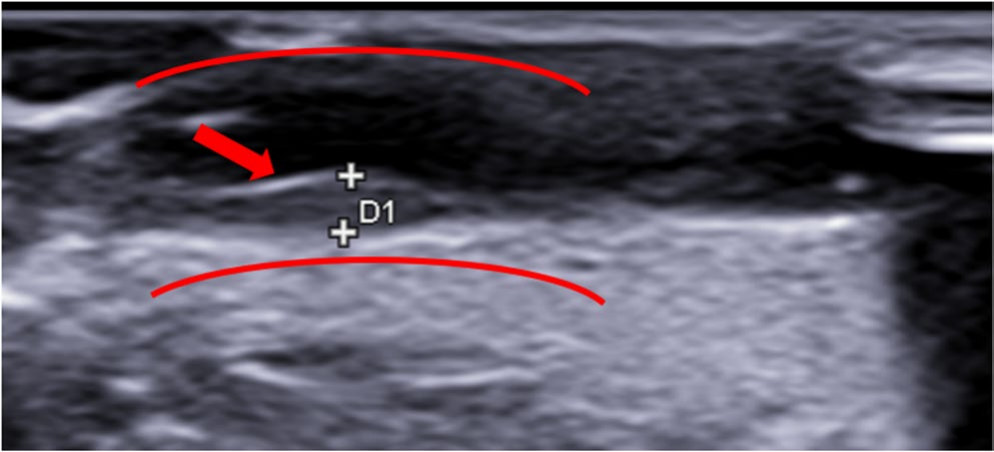
Longitudinal scan showing intimal thickening. The red arrow indicates the site of thickening, which measures 1.2 mm

**Fig. 5:**
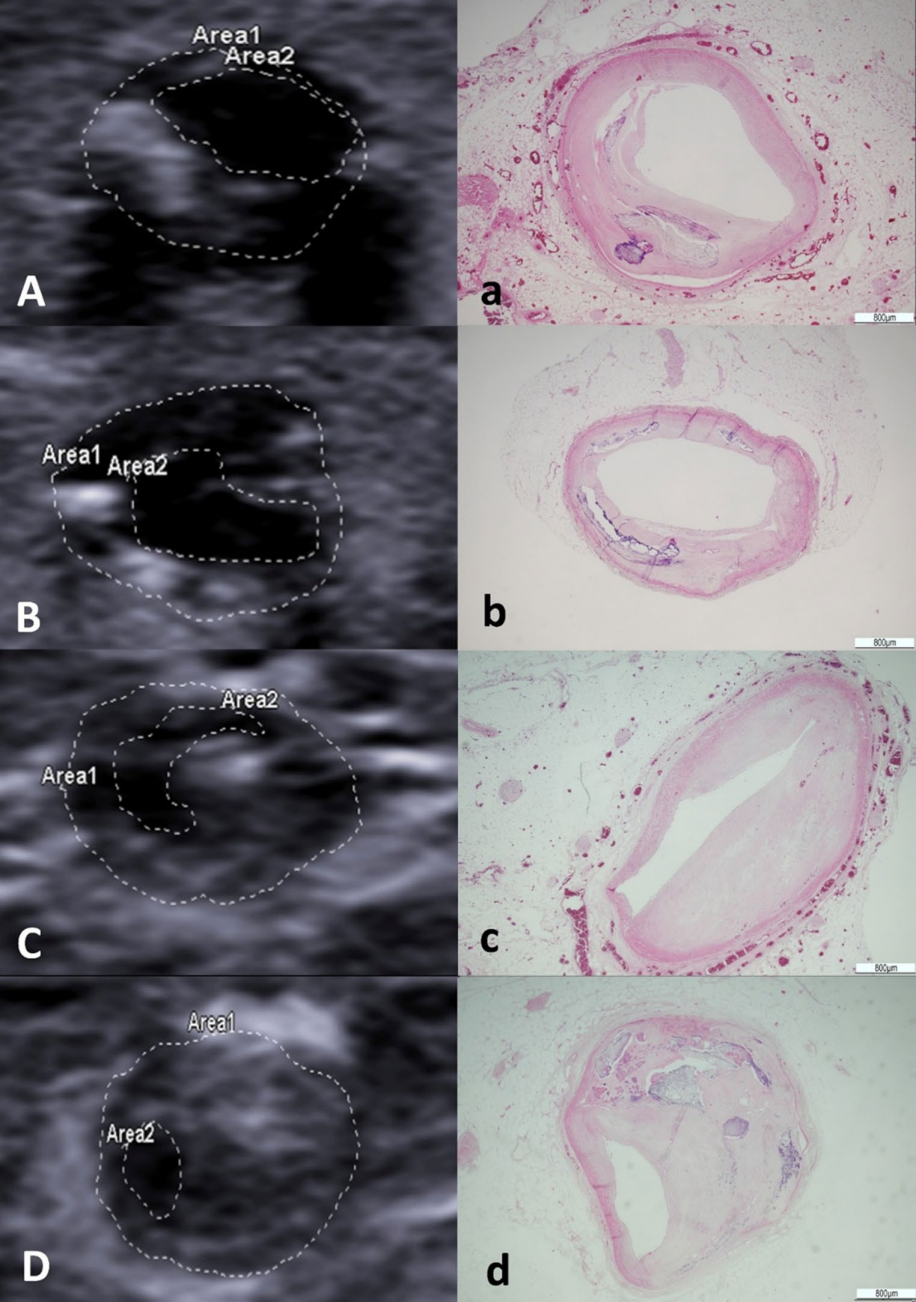
Comparison between US and histology: obstruction of the anterior interventricular artery at 65% (**a**); obstruction of the anterior interventricular artery at 72% (**b**); obstruction of the right coronary artery at 84% (**c**); obstruction of the right coronary artery at 94% (**d**). Area 1 denotes the total cross-sectional area of the vessel, whereas Area2 represents the remaining patent lumen following the obstruction

 The US scans allowed the visualization of the pathological processes. It was possible to appreciate and measure thickening of intima, atherosclerotic plaques and lumen (Fig. 4 and Table 1). Moreover, The eco-structure of atherosclerotic plaques displayed different degrees of echogenicity (Fig. 5)

**Table 1.** Morphometric assessment of main atherosclerotic plaques through US from two different radiologists and at histology. Abbreviations: right coronary artery(RCA), left anterior descending (LAD)

Heart ID	Coronary Artery	Ultrasound Measurement (mm)		Histological Measurement (mm)
		*Radiologist 1*	*Radiologist 2*	
Heart 1	LAD	1.4	1.4	1.25
	RCA	1.5	1.6	1.30
Heart 2	LAD	2.4	2.3	2.10
	RCA	1.6	1.6	1.44
Heart 3	LAD	1.0	1.1	0.94
	RCA	1.1	1.2	1.01
Heart 4	LAD	2.1	2.1	1.87
	RCA	2.4	2.3	2.18
Heart 5	LAD	0.5	0.3	0.20
	RCA	0.2	0.6	0.40
Heart 6	LAD	0.7	0.9	0.90
	RCA	1.1	0.9	0.8

### Pathological examination

During the macroscopic exam, the pathologist confirmed the presence of the major atherosclerotic plaques of the CAs detected with the US, but it was also possible to detect other minor alterations that were not visible at the US, such as fatty streaks. The microscopic assessment of the plaques revealed various grades of vessel occlusion and plaques morphology, and could also well characterize calcium deposits, cholesterol crystals, fibrous caps and their alterations. There was a correspondence between the occlusion detected at the US scan and the assessment of the pathologist for main critical plaques. On the other hand, US and histology display a minor concordance considering smaller noncritical plaques. 

### Functional study

**Fig. 6:**
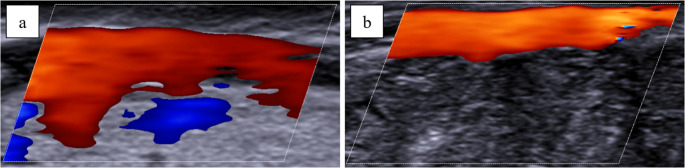
Longitudinal scan of the anterior interventricular artery (**a**) and of the right coronary artery (**b**). Color Doppler ultrasound revealed steady blood flow within both vessels

Color Doppler US scans showed a steady flow (Fig. 6a and 6b) enabling velocity sampling as well.

The minimum velocity recorded was 5.5 cm/s, while the maximum was 82 cm/s. After the transversal cut made on the distal segment of the arteries, higher velocities were recorded, with maximum values of 178 cm/s and 186 cm/s. In smaller vessels, the velocity measurement was more difficult because of the narrower diameter. In fact, in main branches such as the proximal anterior interventricular coronary artery, velocity could be measured without an incision. In narrower segments, however, a measurable flow was achieved only after a transverse cut was made at the vessel's distal end.

**Fig. 7:**
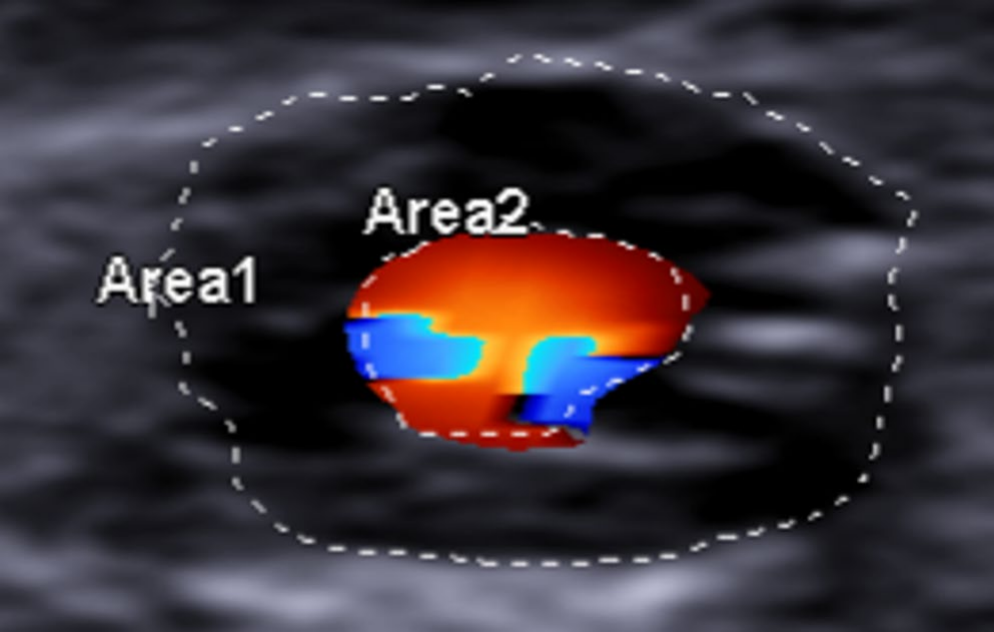
Transverse scan. Color Doppler ultrasound revealed turbulent flow in the vessel segment exhibiting an 83% obstruction due to an atherosclerotic plaque

 In coronary arteries with hemodynamically significant plaques (>75% stenosis [23]), color Doppler imaging showed turbulent flow distal to the lesion, which appeared as a characteristic mosaic of aliased red and blue signals (Fig. 7).

**Fig. 8:**
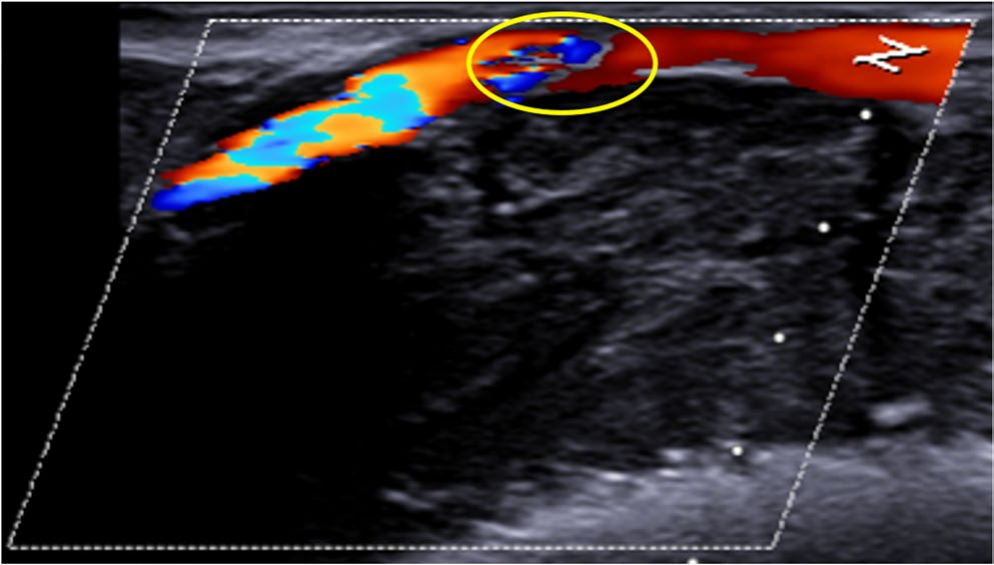
Longitudinal scan. Color Doppler ultrasound revealed turbulent flow within the segment obstructed by the atherosclerotic plaque (yellow oval) as well as in the downstream vascular segment

 The sampling of coronary flow velocities in the boundaries of vessels affected by atheromatic lesions showed variable values, averagely higher, ranging from 28.5 to 210.3 cm/s. Notably, the flow was continuous and nonturbulent upstream of the plaque but became turbulent at the lesion and in the downstream section (Fig. 8).

## Discussion

In cases of SCD, the most widespread post-mortem imaging techniques in forensic medicine are Computed Tomography (CT), Magnetic Resonance Imaging (MRI) and their contrast-enhanced applications [20]. However, in practice, many forensic autopsies are performed in non-specialized centers that lack the appropriate diagnostic instruments. In such scenarios, it can be useful to remove the heart *in toto* for subsequent examination in a more suitable and controlled environment. To support this ex-situ approach, new technologies have been implemented to supplement traditional methods, particularly those that are rapid, non-destructive, and can be performed either on-site or on excised hearts [21, 22, 24–27]. On these grounds, our study aimed to evaluate US as an additional technique for the post-mortem assessment of coronary circulation in ex-situ human hearts. 

 The well-known advantages of US (non-invasiveness, dynamic capabilities, and the ability to evaluate structures from both a morphological and functional perspective) make it a particularly relevant tool for vascular assessment in the clinical setting. Even for the evaluation of CAs in pediatrics, it has often been indicated as a diagnostic method of choice, especially for the detection and study of coronary malformations [5]. On the other hand, in adult subjects, evaluation of coronary circulation through US remains cumbersome because of the limited anatomical access, which would allow visualization of only certain segments of the coronaries, and the loss of signal quality due to the major representation of thoracic tissues (skin, adipose tissue, bones, etc.) interposed between the probe and the coronary arteries. Clearly, these limitations can be overcome in the forensic setting, since the course of the coronaries in the heart is superficial and US coronarography can be performed on ex-situ hearts. This might allow the forensic pathologist to assess the heart with a complete US examination achieving a full access of the coronaries and without the interference of other tissues. Nevertheless, in the absence of active blood flow, the visualization of vessels is cumbersome due to their collapse and US dDoppler technique is not feasible. On the other hand, through active instillation of contrast media in the coronary vessels a simulated coronary circulation can be implemented, and morphological and functional study become feasible.

### Morphological study

Regarding the morphological analysis, in the absence of flow, it was particularly difficult to identify the vessels and nearly impossible to recognize and assess their lumen. The simulation of a post-mortem artificial circulation producing vascular distension allowed a satisfactory morphological evaluation. In particular it was possible to appreciate the coronary circulation and vascular components, such as the vessel walls and ongoing pathological processes (e.g., intimal thickening and atherosclerotic plaques). Although the cardio-pathologist’s examination remains the gold standard investigation for the diagnosis of cardiac deaths, US showed a substantial advantage, namely the possibility, once the pathologies of interest are identified, to perform measurements, such as the thickness of the intimal thickening and the percentage of occlusion of the vessel affected by an atherosclerotic plaque, before dissecting the heart. Moreover, after US evaluation it was possible to focus and thus to optimize the following cardio-pathologic investigation. This is a substantial advantage in the evaluation of cases of suspected cardiac death, since it is often indicated in the literature that atherosclerotic plaques with a luminal stenosis greater than 75% are to be considered a highly probable cause of cardiac death and SCD [19]. Currently, this level of detail is typically only available from invasive examinations in living patients, like coronary angiography. Access to this clinical data allows a forensic pathologist to determine if a specific atherosclerotic lesion was fatal. In such context, the US might represent a significant advantage, which would allow for a rapid, preliminary evaluation of hearts in the autopsy room to quickly understand whether there are pathological processes of interest, and would make it easier for the cardio-pathologist, during the necessary and essential pathological evaluations, to identify the vascular segments affected by the most significant atherosclerotic phenomena for a more targeted assessment.

### Pathological examination 

Macroscopic examination of the major coronary arteries through serial transverse sections confirmed the location of the plaques previously identified by ultrasound (US). Histological analysis was then performed on these atherosclerotic plaques from the left anterior descending (LAD) and right coronary artery (RCA) vessels.

### Functional study

The primary innovation of this US method is the functional evaluation of the coronary circulation. Preliminary results indicate that Doppler US enables the assessment of coronary flow in post-mortem samples, allowing for an evaluation of the hemodynamic consequences of atherosclerosis on a simulated circulation. Repeated measurements of flow velocity revealed marked variability. This is not surprising, as flow velocity depends not only on vessel features (such as lumen patency and wall elasticity) but also on other variables like the infusion pressure and density of the perfusing medium and vascular resistance. On this point, it was observed that sectioning the distal segments of the vessels diminished downstream resistance, causing a marked increase in flow velocity. Furthermore, a reduction in the vessel’s cross-sectional area led to an increase in flow velocity, a phenomenon observed at the boundaries of atherosclerotic lesions obstructing the lumen in our casuistry. Moreover, while the flow in unobstructed vessels was linear and continuous, evaluation upstream and downstream of the obstruction revealed turbulence, mirroring the findings of color Doppler US features in the clinical setting.

### US compared with other post-mortem imaging techniques

Numerous experimental methods have been used to study the heart and coronary circulation [21, 28–35].

In current practice, the primary post-mortem imaging techniques employed in cases of SCD are Computed Tomography (CT), Multiphase Post-Mortem CT-Angiography (MPMCTA), and Post-Mortem Magnetic Resonance Imaging (PMMRI). Standard CT provides a static image of the coronary circulation. MPMCTA is more widely used because it employs a contrast agent for a dynamic evaluation of the circulation. This technique has proven sensitive for assessing both the aorta and smaller-caliber vessels, allowing for the visualization of vascular system alterations [36]. Specifically, MPMCTA facilitates the visualization of coronary arteries and enables the assessment of stenoses, occlusions, and calcifications [37].

PMMRI is used less frequently in SCD cases but is effective for identifying signs of chronic or acute myocardial infarction [38, 39]. An ex-situ PMMRI study on five hearts demonstrated that hyperintense areas were associated with edema or pathological fibers, while hypointense areas correlated with fibrosis, confirming the protocol's effectiveness for ex-situ heart assessment [40]. Therefore, in SCD cases, MPMCTA plays a central role in evaluating the coronary circulation, whereas PMMRI is more relevant for assessing myocardial tissue.

However, these advanced techniques have some limitations. Both CT and MRI scanners represent a substantial initial investment. While this equipment is becoming more widespread in major forensic medicine institutes, in other settings it is shared with clinical departments, which introduces the logistical and financial costs of transferring the body and performing the exams outside of clinical activity. Furthermore, the long acquisition times for PMMRI make the procedure particularly time-consuming.

In this context, post-mortem US presents several practical advantages that make it a compelling complementary tool. First, compared with the aforementioned methods, US is a portable and low-cost device. Furthermore, an ex-situ US examination allows for a real-time imaging evaluation of vessel conditions and plaque morphology within the context of the autopsy itself. US offers the opportunity to guide the targeted opening of suspicious coronary segments. However, the primary limitation of US is its operator-dependent nature, which necessitates either an expert user or, for wider adoption, the implementation of specific training for forensic pathologists.

### Limits and future perspectives

This study has several limitations. Firstly, all cannulated hearts were perfused with a constant infusion pressure and a constant flow rate, whereas physiological vascular flow is pulsatile. Therefore, the constant flow characteristics of the pump preclude a direct comparison of the US findings from this method with data obtained from living patients where blood flow is pulsatile. Future studies should therefore use a pump that simulates pulsatile flow, allowing a better characterization of the functional implications of coronary artery disease in the post-mortem setting.

Furthermore, US is known to be an operator-dependent technique. Examinations performed by different operators yielded inconsistent results. The results, especially regarding the estimation of flow velocity values, showed considerable variability even when performed by the same radiologist. In fact, minimal movements or pressure variations of the transducer could cause signal loss or significantly different flow velocity readings. However, intra- and inter-operator variability could be reduced through the use of more sensitive instrumentation and a transducer of optimal dimensions for the examination of human coronary flow.

Additionally, water was tested as a contrast medium. A future perspective is to use other media with higher viscosity, which could provide more robust results both in terms of morphological analysis and flow evaluation, proximal and distal to the stenosis. A further avenue for future research is the use of gaseous contrast media routinely employed in Contrast-Enhanced Ultrasound (CEUS). These agents, like water, are unlikely to damage tissue, and their potential in this field warrants investigation.

Finally, to compare ex-situ US with other currently available post-mortem imaging techniques that evaluate the coronary circulation, it would be beneficial to conduct a study on a case series in which ante- or peri-mortem coronary angiographic images are available for comparison with post-mortem US images. This comparison would help establish an effective correlation between the anatomical and functional reality of coronary artery disease in life and the findings investigated by post-mortem US.

In conclusion, post-mortem US is a promising technique for the morphological and functional evaluation of coronary pathology, due to its effectiveness in detecting pathological processes and its non-invasive, conservative nature. It could potentially serve as a screening test before the heart undergoes the standard cardiopathological examination.
