# Correction to: Post-mortem ultrasound study of coronary circulation on ex-situ hearts

**DOI:** 10.1007/s00414-026-03846-6

**Published:** 2026-05-25

**Authors:** Simone Santelli, Marcello Ridolfi, Filippo Pirani, Elena Giovannini, Guido Pelletti, Giovanni Cecchetto, Susi Pelotti, Paolo Fais

**Affiliations:** 1 Department of Medical and Surgical Sciences, Unit of Legal Medicine, University of Bologna, Via Irnerio 49, 40126 Bologna, Italy; 2 Pediatric and Adult CardioThoracic and Vascular, Oncohematologic and Emergency Radiology Unit, IRCCS Azienda Ospedaliero-Universitaria di Bologna, Bologna, Italy; 3 Department of Legal Medicine and Forensic Sciences, University of Pavia, Pavia, Italy

**Correction to: International Journal of Legal Medicine (2026) 140:879–888**

10.1007/s00414-025-03683-z

In the original version of the manuscript, the co-authors’ given names and family names were swapped in the final version, except for the corresponding author.

The correct author names are listed below.

Simone Santelli

Marcello Ridolfi

Filippo Pirani

Elena Giovannini

Guido Pelletti

Giovanni Cecchetto

Susi Pelotti

Paolo Fais

The original article has been corrected.

